# Bufalin Induces Reactive Oxygen Species Dependent Bax Translocation and Apoptosis in ASTC-a-1 Cells

**DOI:** 10.1093/ecam/nep082

**Published:** 2011-06-18

**Authors:** Lei Sun, Tongsheng Chen, Xiaoping Wang, Yun Chen, Xunbin Wei

**Affiliations:** ^1^MOE Key Laboratory of Laser Life Science & Institute of Laser Life Science, South China Normal University, Guangzhou 510631, China; ^2^Department of Anesthesiology, The First Affiliated Hospital of Jinan University, Guangzhou 510632, China; ^3^Institutes of Biomedical Sciences, Fudan University, Shanghai 200032, China

## Abstract

Bufalin has been shown to induce cancer cell death through apoptotic pathways. However, the molecular mechanisms are not well understood. In this study, we used the confocal fluorescence microscopy (CFM) to monitor the spatio-temporal dynamics of reactive oxygen species (ROS) production, Bax translocation and caspase-3 activation during bufalin-induced apoptosis in living human lung adenocarcinoma (ASTC-a-1) cells. Bufalin induced ROS production and apoptotic cell death, demonstrated by Hoechst 33258 staining as well as flow cytometry analysis. Bax redistributed from cytosol to mitochondria from 12 to 48 h after bufalin treatment in living cells expressed with green fluorescent protein Bax. Treatment with the antioxidant *N*-acetyl-cysteine (NAC), a ROS scavenger, inhibited ROS generation and Bax translocation and led to a significant protection against bufalin-induced apoptosis. Our results also revealed that bufalin induced a prominent increase of caspase-3 activation blocked potently by NAC. Taken together, bufalin induced ROS-mediated Bax translocation, mitochondrial permeability transition and caspase-3 activation, implying that bufalin induced apoptosis via ROS-dependent mitochondrial death pathway in ASTC-a-1 cells.

## 1. Introduction

Apoptosis is a highly regulated and organized cell death process controlling the development and homeostasis of multicellular organisms that occur under a variety of physiological and pathological conditions [1]. It is characterized by a number of well-defined features including cellular morphological change, chromatin condensation, oligonucleosomal DNA cleavage, translocation of phosphatidylserine (PS) from the inner to the outer leaflet of the plasma membrane and activation of a family of cysteine proteases called caspases [2]. Caspases activation is generally considered to be a key hallmark of apoptosis. Caspase-3, an effector caspase, has been implicated in the execution phase of apoptosis [3].

Bax, a death effector of Bcl-2 family, plays an essential role in the mitochondrial pathway of apoptosis [4, 5]. Bax undergoes a conformational change and translocates from cytosol to the outer mitochondrial membrane in the early step of the apoptotic pathway [4]. Translocation of Bax and Bid to the mitochondria leads to the loss of mitochondrial membrane potential (ΔΨ_*m*_) [4, 6–8].

Reactive oxygen species (ROS) have been identified as important mediators regulating signal transduction [9]. ROS can induce injury in a variety of mammalian cells [10, 11] and play crucial roles in the signaling pathway of drug-induced preconditioning [9]. ROS generation has also been demonstrated following the administration of many mitochondrial-dependent apoptotic stimuli including some chemotherapeutic agents [12]. In many apoptosis models, investigators have reported that ROS generation occurs downstream of the release of cytochrome *c* from the mitochondria [13]. ROS have been reported to be involved in various lung cancer cells apoptosis like lung cancer A549 cells [14] and human non-small cell lung cancer cells (NSCLC) [15]. However, the exact mechanism of ROS-mediated apoptosis is still unclear.

Lung cancer is the leading cause of cancer death throughout the world [16]. For therapeutic purposes, lung cancer is generally divided into small-cell lung cancer (SCLC) and non-SCLC (NSCLC) [17], with the latter constituting major populations [18]. Various approaches for lung cancer treatment including induction of differentiation and apoptosis have been attempted, but conventional chemotherapy and radiotherapy of lung cancer are still of limited effectiveness, necessitating the development of new treatment strategy [15]. It is of paramount importance to discover novel agents with less severe side effects and drug resistance.

Bufalin has been found to induce cell apoptosis in various types of cancer cells, but the precise molecular mechanism is still unclear. Bufalin is the major digoxin-like immunoreactive component of Chan-Su, a traditional Chinese medicine obtained from the skin and parotid venom glands of toad [19]. Bufalin exhibit a variety of biological activities, such as cardiotonic, anesthetic, blood pressure stimulation, respiration and antineoplastic activities. In terms of its anti-tumor activities, bufalin has been demonstrated to induce apoptosis in leukemic cells, human hepatocellular carcinoma and prostatic cancer [20–22]. Induction of apoptosis by bufalin in human tumor cells has been reported to associate with a change of intracellular concentration of Na^+^ ions by inhibiting the Na^+^ and K^+^-ATPase on the cell membrane [23, 24].

The fluorescence resonance energy transfer (FRET) technique has been widely used to study protein-protein interactions in living cells [25, 26]. In order to monitor caspase-3 activation in living cells, Miura *et al.* [25] constructed a FRET probe, SCAT3, which consists of enhanced cyan fluorescent protein (ECFP) as a donor, a mutant of yellow fluorescent protein (Venus) as an acceptor and a linking peptide sequence containing a caspase-3 cleavage (DEVD) between ECFP and Venus. Activated caspase-3 cleaves the linker, thus, effectively reduces the FRET [25]. The FRET technique based on SCAT3 has been widely used to monitor the spatio-temporal dynamics of caspase-3 activation in individual living cells in real time in our previous studies [26].

The purpose of this study was to investigate the molecular mechanism of bufalin-induced apoptosis in human lung adenocarcinoma (ASTC-a-1) cells. In addition to conventional methods, time-lapse fluorescence confocal microscopy (CFM) and emission spectral analysis based on FRET were used to monitor the spatio-temporal dynamics of ROS production, Bax translocation and caspase-3 activation during bufalin-induced apoptosis in living cells.

## 2. Methods

Bufalin was obtained from Alexis (Lausen, Switzerland). Working solutions were prepared by dissolving the compound in 100% ethanol before experiment. The final concentration of ethanol was <0.1% in all experiments. Lipofectamine 2000 and Mitotracker Red were purchased from Invitrogen (Carlsbad, CA, USA). NAC (ROS scavenger), Rhodamine 123 (Rho123) and Hoechst 33258 were obtained from Sigma (St. Louis, MO, USA). Cell Counting Kit (CCK-8) was purchased from Dojindo (Kumamoto, Japan). Annexin V (FITC) apoptosis detection kit was obtained from Bender Medsystems (Vienna, Austria) and Ac-DEVD-AFC was purchased from Alexis (Lausen, Switzerland). z-VAD-fmk, a broad spectrum caspase inhibitor, was purchased from BioVision (CA, USA). 2′,7′-Dichlorodihydrofluorescein diacetate (DCFH-DA) was purchased from Wako Ltd (Osaka, Japan). SCAT3 plasmid was kindly provided by Dr. Miura [25].

### 2.1. Cell Culture and Transfection

ASTC-a-1 cell line was obtained from the Department of Medicine, Jinan University (Guangzhou, China). Dulbecco's modified Eagle's medium (DMEM) was purchased from Gibco (Grand Island, NY, USA). For fluorescence studies, the cells were transferred in 35-mm dish 24–48 h after transfection. The cell line stably expressing SCAT3 was obtained from our previous studies [26]. All the cells were cultured in DMEM supplemented with 10% fetal calf serum with 5% CO_2_ at 37°C in a humidified incubator.

### 2.2. Cell Viability Assay

Cell viability after treatment with bufalin was measured by the CCK-8 assay. ASTC-a-1 cells were suspended at a final concentration of 1 × 10^4^ cells/well and cultured in a 96-well flat-bottomed microplate. After different treatment of cells, CCK-8 (10 *μ*l) was added to each well containing 100 *μ*l of the culture medium and bufalin mixture, and the plate was incubated for 1 h at 37°C. Viable cells were counted by absorbance measurements at 450 nm using auto-microplate reader (infinite M200, Tecan, Austria). All experiments were performed in triplicate on three separate occasions.

### 2.3. Cell Apoptosis Detection

Cell apoptosis detection was performed by fluorescence-activated cell sorting (FACS) analysis using a flow cytometer (FCM, Arla BD, USA) with apoptotic cells being annexin V positive/PI negative. The exposure of PS on the extracellular side of the cell membrane was quantified by annexin V/PI staining. Treated cells in a 6-well plate with a density of 1 × 10^6^ were collected by centrifugation (1200 rpm for 3 min) and washed by PBS. Then cells were stained with 5 *μ*l of annexin V-FITC for 30 min and 10 *μ*l propidium iodide (PI, 10 *μ*g/ml) for 10 min at room temperature. After being screened with 300-mesh sieve, cellular DNA was detected by flow cytometry (FCM) and apoptosis rate was computed.

### 2.4. Morphological Examination for Apoptosis

The cells were grown on the cover slip of a 35-mm chamber. After being treated with bufalin for 48 h in the absence or presence of NAC at 37°C, the cells were washed with PBS three times and incubated with 1-*μ*M Hoechst 33258 for 20 min at room temperature in the dark. The cells were then washed three times with PBS and visualized under a Zeiss fluorescent microscope (Axiovert 200M). The images of Hoechst 33258 were recorded using a digital camera (Nikon, Tokyo, Japan) with 1280 × 1280 pixels resolution.

### 2.5. Confocal Fluorescence Live Monitoring

Fluorescence imaging was performed on a confocal microscope (LSM510/ConfoCor2, Zeiss, Jena, Germany). All quantitative analyses of the fluorescence images were performed with the Zeiss Rel3.2 image processing software (Zeiss). For time-lapse imaging of ROS, culture dishes were mounted onto the microscope stage equipped with a temperature-controlled chamber and was stained with 10-*μ*M DCFH-DA in PBS for 15 min at 37°C. DCFH-DA was cleaved by intracellular esterases and turned to the highly fluorescent 2′,7′-dichlorodihydrofluorescein (DCF) upon oxidation by ROS. The fluorescence images of green fluorescent protein (GFP) and DCF were excited by a 488-nm laser and fluorescence emission was recorded through a 500–550-nm band-pass filter.

### 2.6. Mitochondrial Size Determination

The cells were cultivated on a coverslip of a 35-mm chamber. At indicated times after being treated with bufalin, the cells were washed with PBS three times and incubated with 0.1-*μ*M Mito-tracker Red for 30 min at room temperature in the dark. Then the cells were washed three times with PBS and visualized. Mito-tracker Red was excited by a 633-nm laser and the emitted light was recorded through a 650-nm long-pass filter. For each condition tested the size of 100–200 mitochondria in at least 50 different cells was measured from three independent experiments.

### 2.7. Measurement of Mitochondrial Membrane Potential (ΔΨ_*m*_)

Rho123 was used to evaluate the changes of ΔΨ_*m*_ by FCM. The cells were grown at a density of 1 × 10^5^ cells in 12-well flat-bottomed microtiter plates. After treatment with bufalin for indicated times in the absence or presence of NAC, the cells in 1-ml PBS were stained with 1 *µ*M Rho123 for 30 min in dark at room temperature. The cells were then collected by centrifugation (1200 rpm, 3 min), washed with PBS three times and resuspended in 1-ml PBS. Fluorescence emitted from the Rho123 was detected with a flow cytometer (FACS. Arla BD, USA).

### 2.8. Fluorescence Spectral Analysis Inside Living Cells

ASTC-a-1 cells stably expressing SCAT3 were cultured in the 96-well flat-bottomed microplate for 24 h. The cells were then incubated with 0.1-*μ*M bufalin for 48 h and 1-*μ*M STS for 12 h in the presence or absence of 10-*μ*M z-VAD-fmk or 5-mM NAC. The emission spectra of the SCAT3 were detected by auto-microplate reader (infinite M200, Tecan, Austria). In the meantime, emission spectra of non-transfected cells were obtained as background. The step length of the scanning spectra is 2 nm. The excitation wavelength of SCAT3 was 409–427 nm and the emission fluorescence channel was 454–600-nm band-pass.

### 2.9. Fluorometric Assay for Caspase-3 Activity

For the detection of caspase-3 activity, PBS-washed cell pellets (derived from either the medium or the adherent cells) were resuspended in extract buffer (25-mM HEPES (pH 7.4), 0.1% TritonX-l00, 10% glycerol, 5-mM DTT, 1-mM phenylmethylsulfonyl fluoride, 10-mg/ml pepstatin, and 10-mg/ml Leupeptin) and vortexed vigorously. A 20 *μ*l of the extracts (corresponding to 10% of the sample) were incubated with the caspase-3 fluorogenic substrates Ac-DEVD-AFC at a 100 *μ*M final concentration at room temperature. Caspase-3 activity was measured continuously by monitoring the release of fluorigenic AFC at 37°C. The excitation wavelength of AFC was 400 nm and the emission wavelength was 530 nm using auto-microplate reader (infinite M200, Tecan, Austria).

### 2.10. Protein Extraction and Western Blot Analysis

For western blot analysis, the cells were treated with 1-*μ*M STS for 12 h, 0.1-*μ*M bufalin for 48 h and combined treatment with 0.1-*μ*M bufalin and 5-mM NAC for 48 h. Collected cells were washed with cold PBS and lysed in lysis buffer (50-mM Tris-HCl, pH 8.0, 150-mM NaCl, 1% Triton-100, 1-mM PMSF and protease inhibitor cocktail set I). Protein content was quantitated using the Bradford assay. Equivalent amounts of total protein were resolved by 15% SDS-PAGE and transferred to nitrocellulose membranes (Millipore Co., Billerica, MA, USA) following the conventional protocols. Before being immunoblotted, the membranes were blocked in 5% non-fat milk in TBST buffer (10-mM Tris pH 7.5, 150-mM NaCl, 0.1% Tween 20) for 1 h at room temperature. The rabbit polyclonal anti-caspase-3 (Cell signaling, Cat. no. 9746) was used at a dilution of 1:1000 for overnight at 4°C and secondary anti-rabbit IgG-HRP (Rockland, Gilbertsville, PA, USA) was used at 1 : 3000 for 2 h. Detection was performed using the LI-COR Odyssey Infrared Imaging System (LI-COR, Inc., Lincoln, NE, USA)

### 2.11. Statistical Analysis

The results are expressed as mean ± standard deviation (SD). Statistical analysis was performed with SPSS 10.0 software for multiple comparisons. A *P*-value of <.05 was considered to be statistically significant.

## 3. Results

### 3.1. Bufalin-Induced Cell Apoptosis

**Figure 1:**
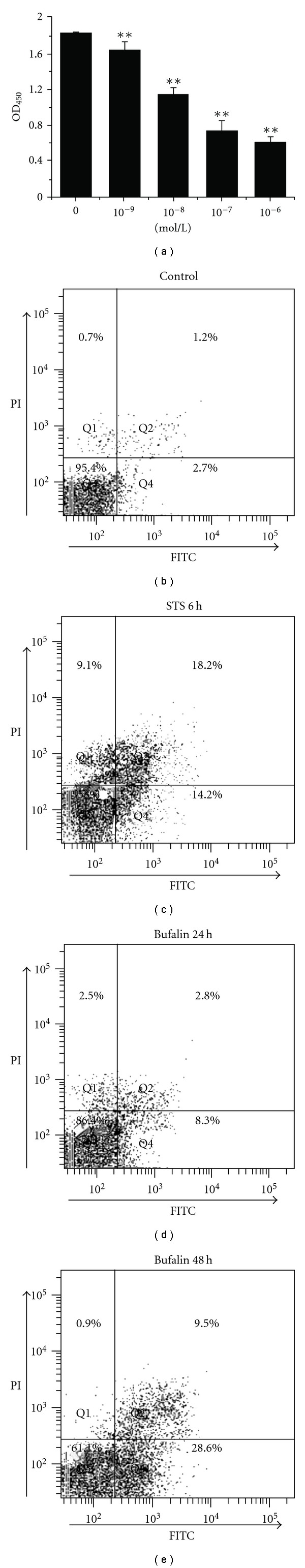
Bufalin-induced cell apoptosis. (a) Inhibition of bufalin on the cell viability at 48 h after bufalin treatment. Cell viability was measured by the CCK-8 assay. **P <.05, compared with control. FCM analysis of apoptosis by annexin V/PI staining for control (b), treatment with STS (C), and treatment with bufalin for 24 h (d) or 48 h (e).

The effect of bufalin on cell viability was assessed using CCK-8. Our data showed that bufalin induced a dose-dependent cell death (Figure 1(a)). The dose of 0.1-*μ*M bulfalin was then adopted in the following experiments for indicated times. To further determine the form of bufalin-induced cell death, we used FCM to analyze the PS alteration and membrane disruption of cells co-stained with annexin V-FITC and PI after bufalin treatment (Figure 1). The cells treated with STS were analyzed as positive control (Figure 1(c)). We found out that bufalin induced a significant increase in FITC fluorescence (Figures 1(d) and 1(e)), while the PI fluorescence in the Q1 area was almost the same as the control (Figure 1(b)), suggesting that bufalin induced apoptotic cell death.

### 3.2. ROS Involvement in Bufalin-induced Cell Apoptosis

**Figure 2:**
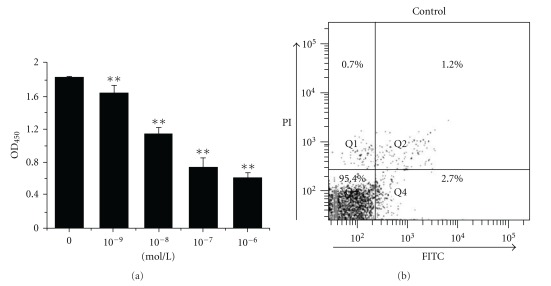
Dynamics of ROS production during bufalin-induced apoptosis. (a) Fluorescence images of DCF inside living cells after treatment with bufalin in the presence or absence of NAC. Scale bar: 10 *μ*m. (b) The dynamical progress of ROS generation corresponding to (A). Data were obtained from three independent experiments.

In order to investigate ROS generation during bufalin-induced apoptosis, the ROS-sensitive vital dye DCF was used to detect the production of ROS in real time by CFM. We found out that bufalin induced a marked increase in DCF fluorescence within 30 min, which was inhibited completely by NAC (Figure 2(a)). The dynamics of the DCF fluorescence corresponding to Figure 2(b) were shown in Figure 2(b). These data showed that bufalin induced rapid ROS generation within 30 min in the cells.

**Figure 3:**
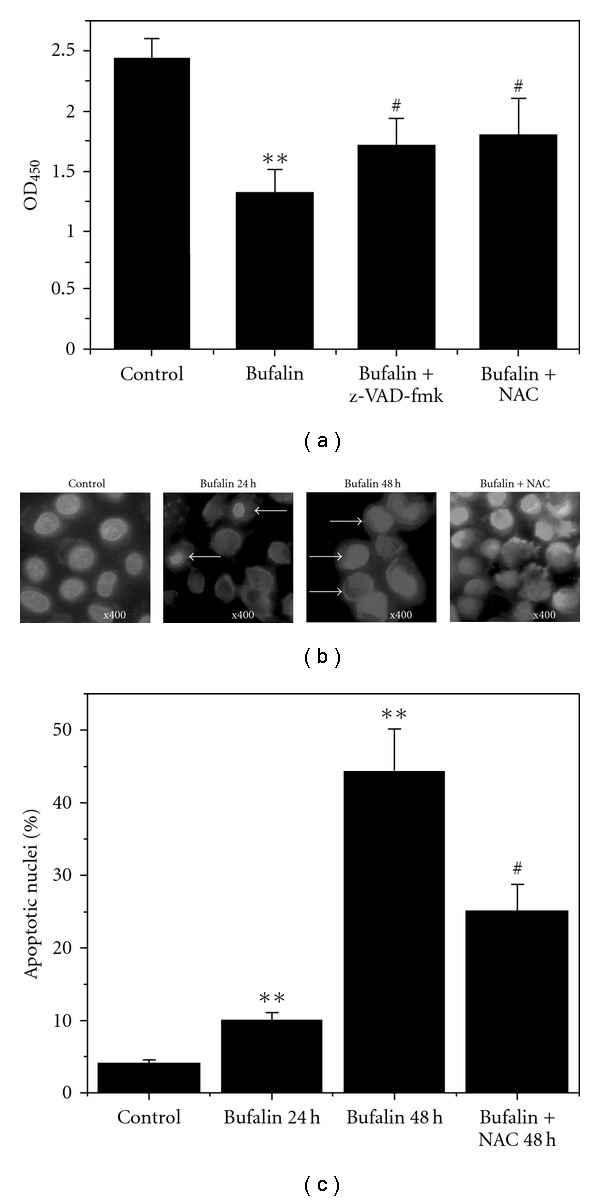
ROS involvement in bufalin-induced apoptosis. (a) Inhibition of z-VAD-fmk or NAC on the cytotoxicity of bufalin. (b) Typical fluorescence images of cells stained with Hoechst 33258. Magnification ×400. (c) Percentage of apoptotic cells according to Hoechst 33258 staining images. Each column represents the average obtained from three independent experiments. **P < .05, compared with control. ^#^ *P* < .05, compared with treatment with bufalin alone.

We next sought to determine whether ROS were involved in bufalin-induced cell apoptosis. Compared with the control, over 50% of cells lost viability at 48 h after treatment with bufalin alone, whereas pretreatment with NAC markedly eliminated the inhibition of bufalin on cell viability (Figure 3(a)). These results showed that bufalin induced apoptosis via ROS-dependent pathway. The results were further confirmed by cellular morphology using Hoechst 33258 staining (Figure 3(b)). The cells treated with bufalin displayed cell shrinkage, chromatin condensation (arrows indicate the nuclei of apoptotic cells) and margination in the nucleus, which were markedly prevented by treatment with NAC (Figure 3(b)). Percentage of apoptotic cells from Hoechst 33258 staining images at 24 h after bufalin treatment and 48 h after bufalin treatment in the absence or presence of NAC are shown in Figure 3(c). Collectively, these results showed that ROS were involved in bufalin-induced cell apoptosis. In addition, pretreatment with z-VAD-fmk showed significant protection to bufalin-treated cells, indicating that caspase family were involved in bufalin-induced apoptosis (Figure 3(a)).

### 3.3. ROS Involvement in Bufalin-induced Bax Translocation

**Figure 4:**
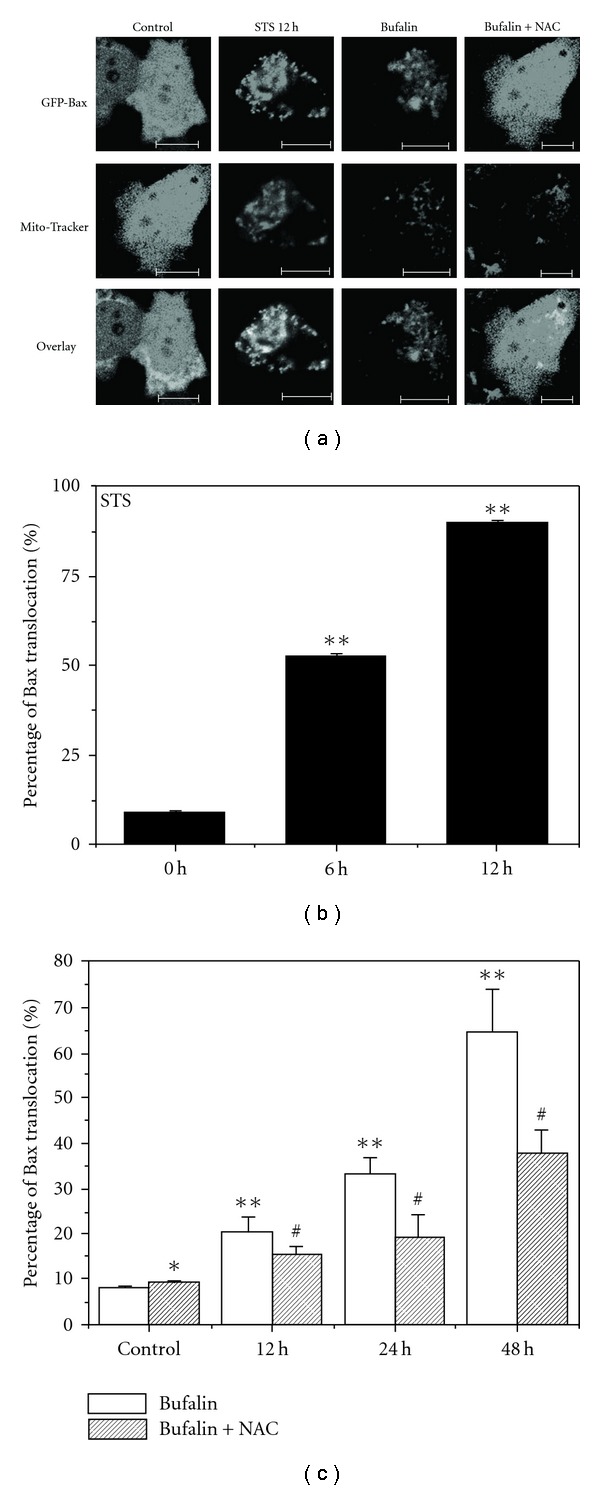
Bufalin induced ROS-dependent Bax translocation. (a) Typical fluorescence images of cells expressing GFP-Bax after different treatments. Mitotracker was used to label mitochondria. Scale bar: 10 *μ*m. (b) Percentage of cells with Bax translocation at 0, 6 or 12 h after STS treatment. (c) Percentage of cells with Bax translocation at 0, 12, 24 or 48 h after bufalin treatment in the absence or presence of NAC. Data were collected in 400–500 cells per treatment in 15–20 randomly selected image frames and from three independent experiments (*n* = 3). Results were presented as mean ± SD. *P > .05, compared with control. **P <.05, compared with control. ^#^ *P* <.05, compared with treatment with bufalin alone.

In some models of mitochondrial-dependent apoptosis, ROS act upstream to cause the activation of Bax or Bak [27]. To explore whether ROS acted upstream of Bax activation in bufalin-induced apoptosis, we used CFM to image the Bax distribution in living cells expressing GFP-Bax after STS or bufalin treatment in the presence or absence of NAC (Figure 4(a)). STS treatment resulted in time-dependent Bax translocation (Figures 4(a) and 4(b)), and bufalin also induced time-dependent Bax translocation, which was markedly inhibited by NAC (Figures 4(a) and 4(c)). The data were collected from 400 to 500 cells per treatment in 15–20 randomly selected image frames from at least three independent experiments. These results implied that ROS mediated the bufalin-induced Bax translocation and apoptosis.

### 3.4. Bufalin-Induced ROS-Dependent Mitochondrial Dysfunction

**Figure 5:**
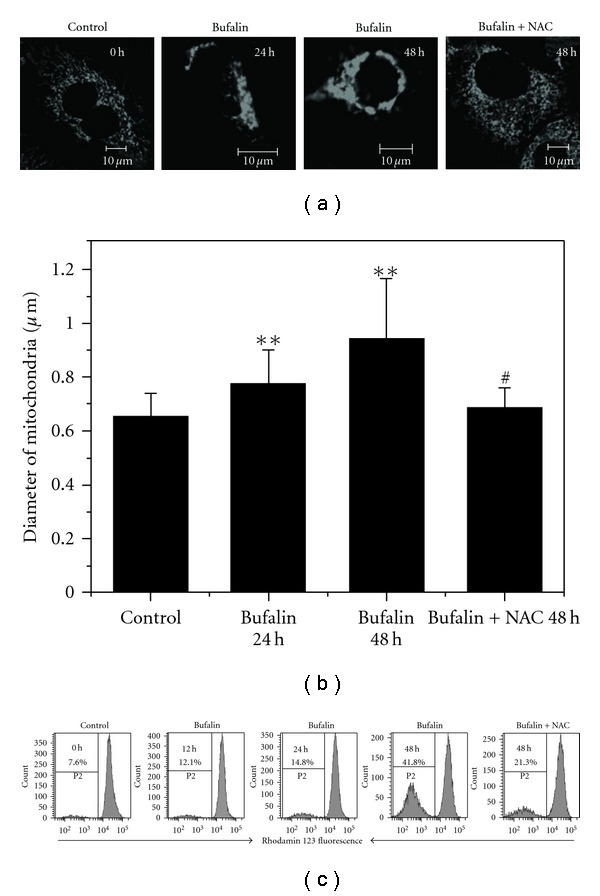
Mitochondrial dysfunction induced by bufalin. (a) Typical fluorescent images of mitochondria under different treatments. The mitochondria in bufalin-treated cells were obviously swollen. Scale bar: 10 *μ*m. (b) Quantification of mitochondrial width under different treatments. **P <.05, compared with control. ^#^ *P* <.05, compared with treatment with bufalin alone. (c) FCM analysis of mitochondrial membrane potential (ΔΨ_m_) loss induced by bufalin. The proportions of cells with reduced Rho123 fluorescence were shown. Experiments were performed in triplicate with similar results.

Since mitochondria play an important role in the apoptotic process, we next evaluated whether bufalin triggered mitochondrial events of apoptosis. One of the mitochondrial events is the ultrastructural changes. Treating the cells with bufalin for 48 h influenced the morphology of mitochondria, which were significantly eliminated by NAC (Figure 5(a)). We also found out that the size of mitochondria gradually increased from 0.65 ± 0.09 to 0.93 ± 0.23 *μ*m after bufalin treatment compared with the control (Figure 5(b)), while the size of mitochondria under combined treatment with bufalin and NAC was 0.68 ± 0.08 *μ*m. Another important mitochondrial event related to apoptosis is the permeability change, which is associated with a drop in mitochondrial membrane potential (ΔΨ_m_). We used FCM to detect the loss of ΔΨ_m_ by measuring the percentage of cells with Rho123 fluorescence under various treatments (Figure 5(c)). The percentage of cells with Rho123 fluorescence after treatment with bufalin alone for 0, 12, 24 or 48 h were 7.6%, 12.1%, 14.8% or 41.8%, respectively, with the latter reducing to 21.3% in the presence of NAC (Figure 5(c)). These results indicated that bufalin induced a time- and ROS-dependent loss of ΔΨ_m_.

In addition, we monitored the dynamics of cytochrome *c* release events inside single living cell co-expressing Cyt-c-GFP and DsRed-mito using time-lapse CFM (LSM510-Meta, Zeiss, Germany). Our data showed that cytochrome *c* was not released from mitochondria to cytosol under bufalin treatment for 24 h, implying that cytochrome *c* was not involved in bufalin-induced apoptosis. We also detect the caspase-9 activation during bufalin-induced apoptosis inside living cells expressing SCAT 9 plasmid using FRET acceptor photobleaching and emission spectral analysis as we did in previous studies [8, 26]. Our data showed that caspase-9 was not activated under bufalin treatment.

### 3.5. Bufalin-Induced ROS-mediated Caspase-3 Activation

**Figure 6:**
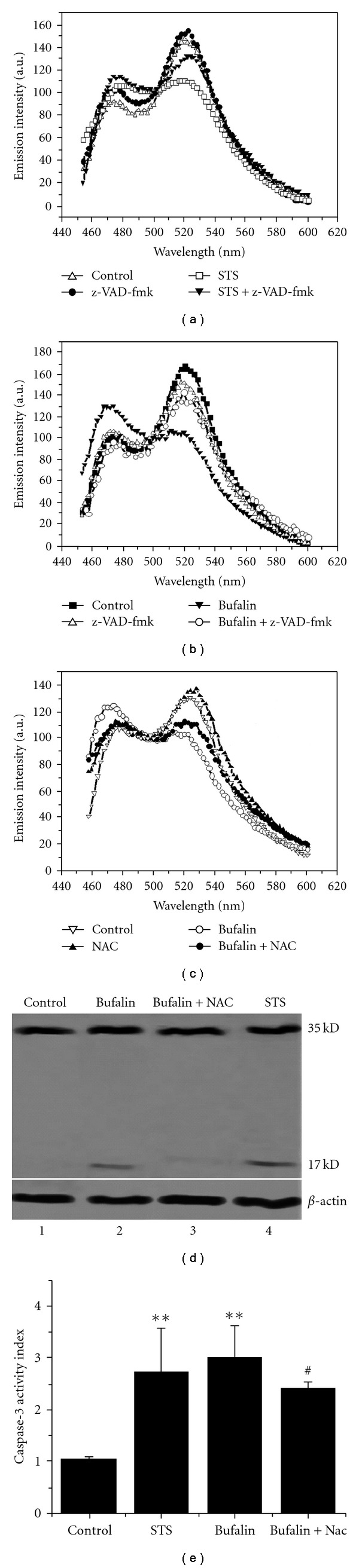
Caspase-3 activation induced by bufalin via ROS-dependent pathway. (a) STS-induced caspase-3 activation. (b) Bufalin-induced caspase-3 activation. (c) ROS-mediated bufalin-induced caspase-3 activation. (d) Western blot assay of ROS-mediated caspase-3 activation induced by bufalin. (e) Fluorometric assay of ROS-mediated caspase-3 activation induced by bufalin.**P <.05, compared with control; ^#^ *P* <.05, compared with treatment with bufalin alone.

Caspase-3 activation was examined during bufalin-induced apoptosis in living cells expressed stably with SCAT3 by monitoring the fluorescence emission spectra of SCAT3, which had been used in our previous study [26]. Bimodal emission peaks at 480 and 524 nm were observed with 430-nm excitation. The strong peak at 524 nm of Venus was due to the FRET between ECFP and Venus in control cells (Figure 6(a)). STS activated caspase-3, which cleaved SCAT3, and then led to a significant decrease at the peak of 524 nm and a corresponding increase at the peak of 480 nm , which was markedly inhibited by z-VAD-fmk (Figure 6(a)). Similarly, the peak at 524 nm completely disappeared at 48 h after bufalin treatment, which was also inhibited by z-VAD-fmk (Figure 6(b)), implying that bufalin activated caspase-3. In order to verify whether ROS were involved in the bufalin-induced caspase-3 activation, we also measured the emission spectra of SCAT3 after combined treatment with bufalin and NAC. NAC significantly attenuated the decrcease at the peak of 524 nm compared with bufalin treatment alone (Figure 6(c)), indicating ROS were involved in the bufalin-induced caspase-3 activation.

Western blot analysis was performed to further confirm bufalin-induced ROS-dependent caspase-3 activation. Caspase-3 was primarily present as the 35-kDa pro-form for the untreated cells (Figure 6(d), lane 1). Following exposure to STS for 12 h, caspase-3 activation was detected by the loss of pro-form and the appearance of processed caspase-3, p17 fragment from cleavage of caspase-3 (Figure 6(d), lane 4). Treatment with bufalin for 48 h showed the same result (Figure 6(d) lane 2). In addition, bufalin-induced caspase-3 activation was blocked by combined treatment with bufalin and NAC (Figure 6(d), lane 3).

We next used AFC to further detect caspase-3 activation during bufalin-induced apoptosis. AFC release is an indicator of caspase-3 activation [28]. The level of caspase enzymatic activity in the cell lysate is directly proportional to the fluorescence signal detected with a fluorescent microplate reader. As shown in Figure 6(e), after incubation with Ac-DEVD-AFC for 1 h, bufalin treatment induced nearly a 3-fold increase in caspase-3 activity compared with control. In contrast, the cleavage of Ac-DEVD-AFC in response to caspase-3 activation was blocked by combined treatment with bufalin and NAC (Figure 6(e)).

Taken together, these results demonstrated that bufalin induced ROS-mediated caspase-3 activation.

## 4. Discussion

In this study, we for the first time used fluorescence techniques and the time-lapse CFM to reveal that bufalin induced apoptosis via ROS-dependent mitochondrial pathway in living ASTC-a-1 cells. Bufalin-induced ROS production reached the maximum within 30 min. Furthermore, ROS mediated Bax translocation and the loss of mitochondrial potential. It has important clinical significance to guide the treatment for lung cancers.

Our results showed that bufalin-induced ROS production was not just generated from mitochondria. Bufalin induced ROS generation throughout the whole cells (Figure 2(a)), which was similar to the results of ROS involvement in lung cancer A549 cells and prostate cell lines apoptosis [14, 22]. However, a number of other reports showed that bufalin inhibited Na^+^–K^+^-ATPase [20, 23, 24], which increased mitochondrial production of ROS [24].

ROS may act upstream of mitochondrial membrane permeabilization and mediate mitochondrial dysfunction in bufalin-induced apoptosis. ROS generation has been reported to occur following mitochondrial outer membrane permeabilization [27]. However, our results showed that ROS acted upstream of the bufalin-induced mitochondria dysfunctions and mitochondrial network dismantling (Figure 5). ROS may promote MPT by causing the oxidation of thiol groups on the adenine nucleotide translocator, which is believed to form part of the MPT pore [29].

One point worthy to be mentioned is that not all Bax translocate to mitochondria in response to bufalin. Some Bax clusters do not co-localize with mitochondria. Instead, they were associated with one or two round mitochondrias (Figure 5(a)). STS treatment led to the accumulation or targeting of Bax to mitochondria as shown in Figure 5(a), which was in accordance with previous studies [30]. It indicates that the mechanism of bufalin-induced Bax translocation is different from that of STS. STS induces Bax insertion into mitochondria membranes [30]. However, bufalin induces Bax translocation to the surface or around the mitochondria.

To investigate which initiator caspase is involved in this apoptosis process, we detected whether caspase-8 was involved in bufalin-induced apoptosis inside living cells expressing the FRET-Bid plasmid using FRET acceptor photobleaching and emission spectral analysis as we did in previous studies [8, 26]. Our data showed that caspase-8 was activated in bufalin-induced apoptosis. Caspase-3 plays an important role in drug-induced cytotoxic apoptosis in NSCLC [31]. Here, we demonstrated that caspase-3 was activated in a ROS-dependent pathway after bufalin treatment (Figure 6). However, caspase-3 was still activated partially if eliminating ROS (Figures 6(c) and 6(e)), implying that there may be some alternative pathways to activate caspase-3 in addition to a ROS-dependent caspase-3 activation pathway in response to bufalin treatment.

**Figure 7:**
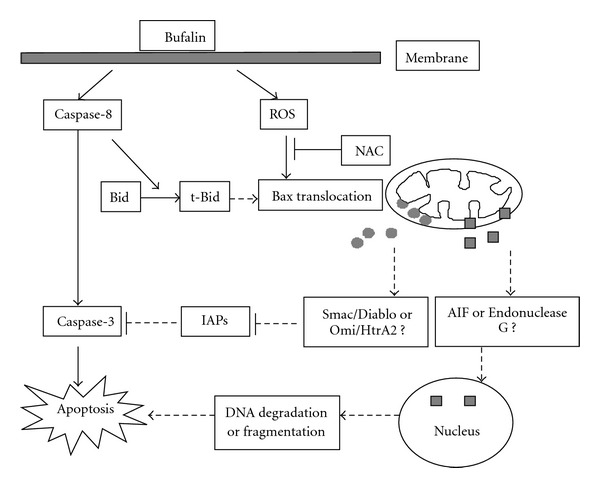
Schematic representation of the bufalin-induced apoptotic pathway in ASTC-a-1 cells. Once engaged by bufalin, two signal pathways are activated. One is the death receptor-mediated pathway and the other is the mitochondrion pathway. Omi/HtrA2: high temperature requirement protein A2; Smac/Diablo: second mitochondria derived activator of caspase/direct inhibitor of an apoptosis-binding protein; AIF: apoptosis inducing factor.

According to the experimental data, we summarize the signaling pathways (Figure 7) related to bufalin-induced apoptosis in ASTC-a-1 cells. Once engaged by bufalin, two signal pathways are activated. One is the extrinsic pathway, in which bufalin induced caspase-8 activation, which subsequently activated caspase-8 directly activates caspase-3. The other is the intrinsic pathway, in which bufalin induced ROS-dependent Bax translocation, loss of mitochondrial membrane potential and caspase-3 activation. Cytochrome *c* release from the mitochondria and caspase-9 activation were not observed after bufalin treatment, suggesting that cytochrome *c* and caspase-9 were not involved in bufalin-induced apoptosis. However, NAC, a ROS scavenger, can partially inhibit caspase-3 activation induced by bufalin (Figures 3(a)–3(c)), implying that Smac/Diablo or Omi/HtrA2 released from the mitochondria antagonize the inhibitors of apoptosis (IAPs) and contribute to activation of caspase-3 [32]. z-VAD-fmk, a broad spectrum caspases inhibitor, did not completely inhibit cell death and caspase-3 activation (Figures 3(b) and 6(b)), indicating that caspase-independent apoptotic pathways may be involved in the bufalin-induced apoptosis, in which AIF or endonuclease G may play a partial role. Bufalin would induce AIF or endonuclease G release from mitochondria to cytosol and translocate to nucleus, causing DNA fragmentation and apoptosis. These imply that bufalin triggers the signaling to induce cell apoptosis via both death receptor-mediated and mitochondrial pathways. We would like to further investigate how other signaling pathways are involved in the bufalin-induced human lung cancer cell apoptosis. We hope our findings on anticancer effects of bufalin will help us improve medical treatment and prevention of human lung cancer.

## Funding

National Natural Science Foundation of China (Grant No. 30670507); the Natural Science Foundation of Guangdong Province (F051001); ‘Shuguang Scholor' (Grant No. 07SG05) of Education Commission; ‘Leading Academic Development Project' (Grant No. B109) of the Science & Technology Commission of Shanghai Municipality.
